# Analysing and quantifying the effect of predictors of stroke direct costs in South Africa using quantile regression

**DOI:** 10.1186/s12889-021-11592-0

**Published:** 2021-08-17

**Authors:** Lyness Matizirofa, Delson Chikobvu

**Affiliations:** 1 Department of Statistics, College of Science, Engineering and Technology, University of South Africa, Florida Campus, 28 Pioneer Avenue, Roodeport, Johannesburg, 1709 South Africa; 2 Department of Mathematical Statistics and Actuarial Science, Faculty of Natural and Agricultural Sciences, University of the Free State, P.O. Box 339, Bloemfontein, South Africa

**Keywords:** Direct costs, Stroke, Risk factors, Quantile regression models, South Africa, Predictors

## Abstract

**Background:**

In South Africa (SA), stroke is the second highest cause of mortality and disability. Apart from being the main killer and cause of disability, stroke is an expensive disease to live with. Stroke costs include death and medical costs. Little is known about the stroke burden, particularly the stroke direct costs in SA. Identification of stroke costs predictors using appropriate statistical methods can help formulate appropriate health programs and policies aimed at reducing the stroke burden. Analysis of stroke costs have in the main, concentrated on mean regression, yet modelling with quantile regression (QR) is more appropriate than using mean regression. This is because the QR provides flexibility to analyse the stroke costs predictors corresponding to quantiles of interest. This study aims to estimate stroke direct costs, identify and quantify its predictors through QR analysis.

**Methods:**

Hospital-based data from 35,730 stroke cases were retrieved from selected private and public hospitals between January 2014 and December 2018. The model used, QR provides richer information about the predictors on costs. The prevalence-based approach was used to estimate the total stroke costs. Thus, stroke direct costs were estimated by taking into account the costs of all stroke patients admitted during the study period. QR analysis was used to assess the effect of each predictor on stroke costs distribution. Quantiles of stroke direct costs, with a focus on predictors, were modelled and the impact of predictors determined. QR plots of slopes were developed to visually examine the impact of the predictors across selected quantiles.

**Results:**

Of the 35,730 stroke cases, 22,183 were diabetic. The estimated total direct costs over five years were R7.3 trillion, with R2.6 billion from inpatient care. The economic stroke burden was found to increase in people with hypertension, heart problems, and diabetes. The age group 55–75 years had a bigger effect on costs distribution at the lower than upper quantiles.

**Conclusions:**

The identified predictors can be used to raise awareness on modifiable predictors and promote campaigns for healthy dietary choices. Modelling costs predictors using multivariate QR models could be beneficial for addressing the stroke burden in SA.

## Background

Stroke is the second leading cause of death and the third leading cause of long-term disability worldwide [1]. This imposes a huge economic burden [2]. Further, non-communicable diseases (NCDs) are currently accounting for 74% of all deaths and long-term disability. Most of these deaths (8.9 million) are due to cardiovascular diseases such as ischemic heart disease and stroke [3]. More than 80% of the stroke burden occurs in low and middle-income countries, and reliable data on stroke epidemiology and modelling predictors of stroke direct costs are scarce in these settings [4]. The burden of NCD including stroke was also found to be on the rise in Africa and other low and middle-income country (LMIC) settings [5]. In Sub-Saharan Africa (SSA) which includes SA, the burden is on the rise owing to increasing incidences. SSA has the highest stroke incidence of 316/ 100,000 person-years [1]. Moreover, there are young stroke patients in SSA, resulting in a greater number of years of potential productivity and years of life lost (YLL). The high social and economic burden of stroke calls for effective strategies for prevention, treatment, and rehabilitation in SSA [6]. Even though stroke can be prevented and treated, it remains one of the biggest cause of death and long-term disability globally [1]. Stroke is increasingly becoming a major public health issue among adults, especially in developing countries like SA [7]. In SA, stroke is the second highest cause of mortality after HIV/AIDS and is among the top ten leading cause of long-term disability [8], accounting for 25,000 deaths a year and 95,000 years lived with disability (YLD) [8]. Also, the crude stroke mortality was found to be 114 per 100,000 person-years and contributed to 8.7% years lived with disability (YLDs) in the Agincourt sub-district rural area in SA [8]. The crude stroke incidence of 244/ 100,000 person-years was also quoted for SA [9]. Mudzi et al. [10] showed that 25.5% of stroke patients die within three months and 38% within 12 months of hospital discharge in SA. Literature indicates that numerous studies have been focusing on the indirect costs of stroke in different nations including SA. Stroke costs can be classified as direct or indirect costs with indirect costs including death and disability. The majority of studies [1, 11, 12] conducted to examine predictors of indirect costs in several countries used statistical models which might not be optimal to inform targeted cost-effective policies and early interventions because they focus solely on the conditional mean of the response variable distribution. Thus, ordinary linear regression models could not give enough information about the underlying associations, and is not robust to statistical outliers and lacks flexibility in analysing the predictors of stroke direct costs [13]. Modelling the mean as in ordinary linear regression models could miss critical aspects of the relationship that may exist between stroke direct costs and its predictors, especially in the presence of skewed data as is usually the case with cost of illness data [14].

However, not enough is known about modelling predictors of stroke direct costs in SA using the quantile regression (QR) statistical technique. This paper addresses important gaps in the stroke disease literature, utilising data collected between January 2014 and December 2018 in SA. To fill these gaps, this study aims to estimate stroke direct costs, identify and quantify modifiable and non-modifiable predictors of stroke direct costs through QR analysis. The information generated from this study is intended to guide the planning of health services for stroke prevention and management in SA.

## Methods

### Data

Data were collected between January 2014 and December 2018. The study sites consist of the nine provinces of SA with an estimate of the mid-year population of 57.73 million [17]. There are approximately 407 public and 203 private hospitals in SA [18]. This study randomly selected 55% of the 203 private hospitals in all provinces and 45% of the 407 public hospitals were randomly selected across the nine provinces of SA. A stratified probability sampling technique was used to calculate the proportions accordingly. The strata being public and private hospitals. Thus, study data were retrieved from 183 public and 112 private hospitals, making a total of 295 hospitals. Although most South Africans use public hospitals, many of these institutions did not capture good quality pertinent variables while private hospitals were doing so. The proportions used were based on the availability of study variables in the public and private hospital databases, and the total number of private and public hospitals in SA. Therefore, 55% of the data were retrieved from private hospitals and 45% from public hospitals.

According to the South African National Department of Health [18], SA private and public health systems exist in parallel [18]. The majority of the population use public hospitals, despite being chronically under-funded and under-staffed whilst 20% of the population use private hospitals [18]. Further, the National Department of Health manages 10 major teaching hospitals.

A validated data retrieval sheet was used to retrieve study data. Patients’ medical records were reviewed to elicit all medical healthcare services consumed during stroke hospitalisation and out of the hospital. The data retrieval sheet was formulated with all the study variables which include; mode of admission, that is inpatient or outpatient, stroke risk factors that are non-modifiable, and those that are modifiable, and direct costs. The variable type of hospital, that is private or public hospital was anonymous for ethical reasons which meant there was no variable specifying type of hospital admitted, as agreed upon in advance. The study hospitals were sampled from the nine provinces of SA, namely Gauteng, KwaZulu-Natal, Western Cape, Eastern Cape, North West, Free State, Limpopo, Mpumalanga, and Northern Cape. The case managers for the sampled hospitals assisted with data retrieval. The total number of stroke patients was 35,730.

### Study variables

#### Dependent variable

The outcome variable was stroke total direct costs measured on a continuous scale over five years. Inflation was ignored over this short period. Inflation in South Africa was low over the period. Evidenced by figures reported in a Stats SA [15] reports for consumer price index 2014–2018. The inflation rate for 2014 was (6.09%), 2015 (4.58%), 2016 (6.35%), 2017 (5.27%) and 2018 (4.62%).

#### Independent/explanatory variables

This study considered several explanatory variables based on the literature on factors influencing stroke direct costs, especially in developing countries. Factors considered include modifiable, non-modifiable and direct medical costs items.

Diabetes was defined as a fasting glucose concentration of greater than 7.0 mmol/L, cholesterol was defined as fasting cholesterol concentration of at least 5.2 mmol/L, high-density lipoproteins (HDL) cholesterol at least 1.03 mmol/L, and low-density lipoproteins (LDL) cholesterol of at least 3.4 mmol/L. Whilst hypertension was defined with cut off of 140/90 mmHg for up to 72 h and heart problems were defined as current atrial fibrillation, heart failure, ischemic heart disease, and valvular heart diseases [1]. The modifiable risk factors of stroke coded as 0 = no, if the measurement is below the defined value of interest and 1 = yes, if the measurements exceeded the study definition. The race variable in SA is categorised as whites, blacks, coloureds, Indians and Asians. This study combined Indians, and Asians to be one racial group because of the lower numbers.

Direct medical costs were defined as the medical cost of managing stroke disease, the direct medical costs include (diagnosis, inpatient/ hospitalisation and outpatient care, chronic medication, radiological examination computed tomography (CT) or magnetic resonance image (MRI), allied services such as physiotherapy and speech therapy, physician, neurologist, clinical treatment and laboratory examination cost). All such information was collated over the five years. Direct costs include all stroke patients that are suspected of or have confirmed stroke.

### Estimation of direct costs

Direct costs of stroke were estimated by using a prevalence-based approach. The prevalence-based approach estimates the costs of all stroke cases in a given study period [16]. The study direct costs include all medical costs for stroke disease incurred within the five-year study period. These costs included hospitalisation/inpatient care costs, diagnostic costs, outpatient costs, and prevention costs. Direct costs of patient transport were not included. Productivity losses as a result of time off work due to illness or due to caring for a stroke patient were also not included as these are indirect costs. Thus, this study estimated the total stroke direct costs by taking into account the direct costs of all stroke patients admitted during the five-year study period. The cost of an inpatient stay was calculated as documented in the Provincial Gazette, Department of Health (2015). The per-item cost of inpatient stays included hospital bed cost, health professional services e.g. nursing services, physicians, and therapists’ costs. The hourly costs of specialists were obtained from the department of health salary scales. The amount of time by physicians, physiotherapists, occupational, and speech therapists per patient per day was taken from the Provincial Gazette. Also, besides the total direct treatment costs for inpatient care, diagnostic visits and tests, and surgical treatment were estimated by multiplying the calculated number of incident stroke cases with the relevant treatment costs. Based on the department of health gazette, the frequency of outpatient visits was assumed over the year for each patient, after being discharged from the hospital. The unit cost of the stroke outpatient clinic was taken from the gazette. The chronic medication costs were estimated from private and public hospital pharmacies.

### Statistical analyses

Data were analysed using descriptive statistics expressing continuous variables through the mean with their corresponding coefficient of skewness. Categorical variables were presented as frequencies and their associated percentages. In this paper, two models were used, ordinary least squares (OLS) and quantile regression. QR is a modelling technique which commonly use the median regression and estimate the conditional quantiles whilst OLS method focus on the conditional mean [13, 19]. QR analysis was employed to assess the effect of modifiable and non-modifiable risk factors on stroke direct costs. Modelling of stroke direct costs was done to develop a predictive and a descriptive model. Thus, QR leads to more comprehensive results because of its ability to assess the effect of each predictor at any part of the stroke direct costs distribution, whereas linear regression can model only the conditional mean of the stroke direct costs. Since costs data has a skewed distribution, QR analysis was employed because it allows for the study of relationships for extreme (low or high) costs [13]. To examine the effects of predictors at different points of the distribution of stroke direct costs, this study used a QR model developed by Aheto [13].

The response variable of interest is stroke direct costs denoted by *Y,* and *X* is a vector of explanatory variables.

We can model the *τ* quantile of *Y* conditional on *X*_*i*_ = (*x*_*i*0′_*x*_*i*1′_*x*_*i*2′_…. . *x*_*ip*_) using the QR model given as ${Q}_{y_i\mid {X}_i}\left(\tau |{X}_i\right)$ = ${x}_i^T{\beta}_{\tau }$ (1).

**Fig. 1:**
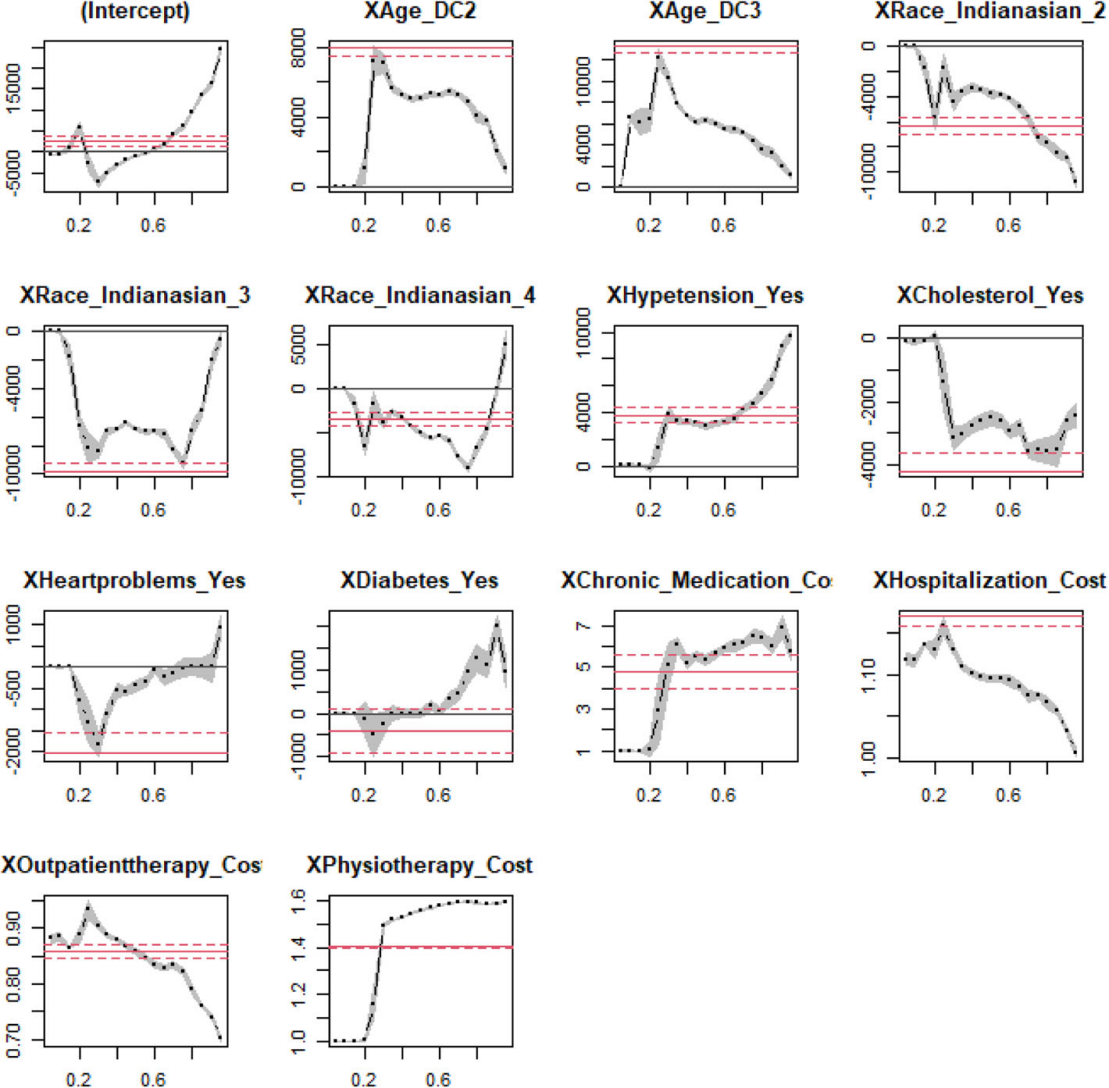
Quantile regression plot of predictors effect on quantiles of stroke direct costs distribution. **Note:** XAge_DC2 = Age group 55–75 years, XAge_DC3 = Age group 76–98 years, XIndianasian_2 = Indian/Asian-race, XIndianasian_3 = Black race, XIndianasian_4 = Coloured race

Where, ${Q}_{y_i\mid {X}_i}$ (*τ*| *X*_*i*_) is the conditional *τ*^*th*^ quantile outcome given *x*_*i*_, *τ* ∈ (0, 1) is the *τ*^*th*^ quantile of the outcome variable stroke direct costs. The variable ${X}_i^T={\left({x}_{i0\prime }{x}_{i1\prime }{x}_{i2\prime}\dots ..{x}_{ip}\right)}^T$ is the vector of covariates for each individual *i and* ${\beta}_{\tau}^T$ = (*β*_*τ*0_, *β*_*τ*1_, *β*_*τ*2_, …*β*_*τp*_)^*T*^ is the vector of (p + 1) regression coefficients at the known *τ*, and *τ* is the 0.1, 0.25, 0.50, 0.75, and 0.95 quantiles. The formulation in (1) permits the modelling of two or more quantiles of stroke direct costs simultaneously while adjusting for the observed covariates [13]. Multivariate QR models were fitted to the stroke direct costs data. The results in Table 4 are graphically displayed in Fig. 1. The quantile plots of predictor effects on the quantiles of stroke direct costs in the model permit visual examination of the predictor effects on each quantile. For analysis, IBM SPSS Version 26 was used to conduct frequencies on demographic and lifestyle characteristics of stroke patients and the R statistical software version 4.0.4. was used for QR analysis of stroke costs. The QR coefficient estimates for the stroke direct costs were also produced with the aid of R statistical package namely quantreg written by Aheto [13] and Koenker [19]. The results from the model were used to examine the covariate effects on each quantile.

## Results

The main demographic characteristics of stroke patients are shown in Table 1. A total of 35,730 stroke patients were retrieved from the patient admission records. There were slightly more females (50.8%) than males. Most patients were whites (34.3%) followed by blacks (29.6%) and fewer coloureds (14.6%). The remainder being Indian/Asian. The average age at the stroke onset was 54.3 years. The incidence of stroke has shown a trend towards relatively younger people in SA with an average age of 54.3 years. A large proportion of patients were relatively young evidenced by 54.5% of patients aged 18–54 years. On average patients were hospitalised for nearly 20 days. The three most common modifiable risk factors were diabetes (62.1%), hypertension (55.3%), and heart problems (54.4%).

**Table 1:** Demographic characteristics of stroke patients and possible predictors (*n* = 35,730)

Categorical variables	n (%)
**Gender**	
Male	17,585 (49.2)
Female	18,145 (50.8)
**Age (in years)**	
18–54	19,474 (54.5)
55–75	10,446 (29.2)
76–98	5810 (16.3)
**Race**	
Black	10,560 (29.6)
White	12,243 (34.3)
Indian/Asian	7724 (21.6)
Coloured	5203 (14.6))
**Hypertension**	
Yes	19,756 (55.3)
No	15,974 (44.7)
**Cholesterol**	
Yes	16,923 (47.4)
No	18,807 (52.07)
**Heart problems**	
Yes	19,453 (54.4)
No	16,277 (45.6)
**Diabetes**	
Yes	22,183 (62.1)
No	1354 (37.9)
**Continuous variables**	**Mean**
Age	54.3 years
Length of stay in hospital (LOS)	19.78 days

The cost figures are summarised in Table 2. Direct costs include medication cost, physiotherapy cost, speech therapy cost, outpatient cost and inpatient/hospitalisation costs for all stroke patients and the total direct costs were (R7.3 trillion over the 5 years). The mean total medication cost per patient was R65 680.02 over the 5 years. The total inpatient care costs (R2.6 billion per 5 -year period) were significantly higher among females with hypertension, cholesterol, heart problems, and diabetes. The average cost of speech therapy was approximately (R329.1 million per 5- year period). There were no patients with zero costs, see Table 2. The mean outpatient cost was relatively low (R33 761.27 per 5-year period). The major drivers for direct costs in SA were inpatient care cost (35%), medication cost (32%), and physiotherapy cost (32%). Further, study findings also show that stroke direct costs data were not normally distributed as indicated by the coefficient of skewness greater than zero. Thus, stroke direct cost data was skewed to the right tail of the cost distribution.

**Table 2:** Direct costs of stroke in South Africa for the period 2014–2018 (Costs in South African Rands)

Cost items	N	Estimated cost	%	Average cost	Skewness coefficient
Medication cost	35,730	2,346,747,100	32	65,680.02	1.178
Physiotherapy cost	35,730	2,348,398,831	32	65,726.25	2.096
Speech-therapy cost	35,730	1,175,724,668	16	32,905.81	2.191
Outpatient cost	35,730	1,206,290,175	16	33,761.27	0.837
Inpatient cost	35,730	2,594,616,496	35	72,617.31	1.907
**Total direct costs**	**35,730**	**7,347,348,497,641**		**205,282.67**	

Table 3 depicts positive intercept for the ordinary least squares linear regression model, entailing that for our basis, a relatively young white male without hypertension, cholesterol, heart problems, and diabetes, with no known other costs and was not hospitalised incurs total stroke direct costs of R 488 (for fixed/admin costs, etc.). The effect of non-modifiable on stroke direct costs is positive and significantly stronger except for the coloured race which is insignificantly different from the basis. The positive coefficients entail that the female gender, higher age groups, other races (Indian/Asians, and blacks) increase stroke direct costs when compared to the basis. Furthermore, modifiable predictors of stroke such as hypertension, cholesterol, heart problems, and diabetes increase stroke direct costs. The effect of diabetes on stroke direct costs are bigger than the effect of other modifiable predictors. Also, the inpatient care effect on stroke direct costs is bigger than the effect of the other cost items. The positive and significant coefficient for inpatient care, outpatient care, medication, physiotherapy, and speech-therapy cost entails that for every one-unit increase for any cost item, stroke direct costs increases by the amount of the coefficient shown in Table 3.

**Table 3:** Results of ordinary least squares linear regression analysis for predictors of stroke direct costs

Variable	Coefficient (***β***_***j***_)	95% CI for ***β***_***j***_	***P***-value
Intercept	0.488	(0.450,0.490)	< 0.0001
Age group 55–75 years	0.799	(0.698,0.809)	< 0.0001
Age group 76–98 years	1.724	(1.700,1.735)	< 0.0001
Female-gender	1.306	(1.300,1.310)	< 0.0001
Indian/Asian-race	1.495	(1.417,1.505)	< 0.0001
Black-race	1.837	(1.800,1.840)	< 0.0001
Coloured-race	2.169	(−2.100,2.209)	0.987
Hypertension-yes	1.152	(1.100,1.202)	< 0.0001
Cholesterol-yes	0.588	(0.549,0.608)	< 0.0001
Heart problems-yes	1.203	(1.199,1.215)	< 0.0001
Diabetes-yes	2.207	(2.204,2.210)	< 0.0001
Chronic medication-cost	1.236	(1.137,1.240)	< 0.0001
Hospitalisation-cost	2.999	(1.980,3.000)	< 0.0001
Outpatient-cost	1.676	(1.566,1.680)	< 0.0001
Physiotherapy-cost	2.090	(1.600,1.706)	< 0.0001
Speech therapy-cost	0.987	(2.000,2.109	< 0.0001

**Note:*β***_***j=***_**coefficient, 95%CI = 95% confidence interval**

Table 4 shows positive intercepts for the five QR models, implying that for our basis, a relatively young white male without hypertension, cholesterol, heart problems, and diabetes, with no known other costs and was not hospitalised incurs total stroke direct costs of R 1, 150 (for fixed/admin costs, etc.) in the 10th quantile. The other intercepts can be interpreted similarly. The quantile regression results show significant differences between lower and upper quantiles. The magnitude of the association between non-modifiable and stroke direct costs fluctuate across all the quantiles. The effect of non-modifiable on stroke direct costs is positive and significantly stronger at the 75th and 95th quantiles except for the coloured race which is insignificantly different from the basis at the 10th quantile. The positive coefficients entail that the female gender, higher age groups, other races (Indian/Asians, and blacks) increase stroke direct costs when compared to the basis. Moreover, modifiable predictors of stroke such as hypertension, cholesterol, heart problems, and diabetes increase stroke direct costs across all the quantiles. The effect of cholesterol and heart problems on stroke direct costs are bigger at the 10th quantiles with less effect at the upper quantiles, whilst diabetes effect on stroke direct costs are bigger at the lower and upper quantiles and less effect at the 50th quantile. Hypertension costs are more or less uniform across all quantiles. The effect of inpatient care on stroke direct costs is bigger at the 10th quantile. However, other cost items have a bigger effect on stroke direct costs at the 95th quantile. The positive and significant coefficient for inpatient care, outpatient care, medication, physiotherapy, and speech-therapy cost entails that for every one-unit increase for any cost item, stroke direct costs increases by the amount of the coefficient. The parameters, in this quantile regression model, including modifiable, non-modifiable, and cost items predictors are positive and significant, thus confirming their greater impact on stroke direct cost distribution when compared with the basis.

**Table 4:** Results of Multivariate quantile regression analysis for predictors of stroke direct costs

Variables	Quantiles ***τ***									
	***τ =*** 0.1		***τ =*** 0.25		***τ=***0.5		***τ=*** 0.75		***τ =*** 0.95	
	***β***_***j***_	**95% CI of*β***_***j***_	***β***_***j***_	**95% CI of*β***_***j***_	***β***_***j***_	**95% CI of*β***_***j***_	***β***_***j***_	**95% CI of*β***_***j***_	***β***_***j***_	**95% CI of*β***_***j***_
Intercept	1.152***	(1.150,1.155)	1.702***	(1.601,1.721)	2.740***	(2.709,2.745)	2.870***	(2.670,2.900)	1.587***	(1.580,1.600)
Female-gender	1.020***	(1.000,1.300)	2.100***	(1.999,2.345)	1.999***	(1.980,2.000)	0.987***	(0.897,1.12)	2.981***	(2.890,3.008)
Age group 55–75 years	1.217***	(1.200,1.220)	1.113***	(1.110,1.118)	1.563***	(1.556,1.600)	1.946***	(1.941,1.956)	1.008***	(1.000,1.113)
Age group 76–98 years	1.531***	(1.430,1.630)	1.804***	(1.800,1.987)	1.738***	(1.730,1.745)	1.940***	(1.899,1.989)	1.227***	(1.117,1.312)
Indian/Asian-race	2.000***	(0.999,2.10)	1.093***	(1.090,1.246)	1.738***	(1.678,1.741)	1.357***	(1.346,1.455)	1.880***	(1.788,1.908)
Black-race	1.551***	(1.457,1.601)	2.500***	(2.167,2.560)	1.986***	1.980,2.100)	1.984***	(1.982,1.999)	1.315***	(1.301,1.412)
Coloured-race	2.698	(−2.598,2.708)	2.470	(−2.430,2.500)	1.476	(−1.46,1.56)	3.423	(−3.404,3.523)	1.553	(−1.550,1.555)
Hypertension-yes	3.249***	(3.200,3.250)	2.157***	(2.100,2.200)	3.419***	(3.314,3.519)	2.528***	(2.428,2.556)	3.230***	(3.123,3.340)
Cholesterol-yes	2.511***	(1.519,2.545)	2.612***	(2.610,2.810)	2.510***	(2.455,2.567)	2.257***	(2.250,2.307)	1.049***	(1.044,1.056)
Heart problems-yes	3.149***	(3.129,3.250)	2.418***	(2.319,2.420)	2.981***	(2.980,3.100)	3.201***	(3.199,3.389)	2.295***	(2.195,2.305)
Diabetes-yes	4.213***	(4.205,5.200)	3.214***	(3.115,3.250)	2.846***	(2.840,2.855)	2.589***	(2.458,2.609)	3.553***	(3.500,3.633)
Hospitalisation-cost	4.189***	(4.109,4.190)	1.989***	(1.900,2.109)	1.000***	(0.899,1.201)	1.283***	(1.183,1.294)	2.788***	(2.688,2.808)
Outpatient-cost	1.000***	(0.998,1.100)	1.715***	(1.700,1.812)	1.936***	(1.914,1.956)	2.415***	(2.399,2.546)	1.607***	(1.606,1.610)
Medication-cost	1.486***	(1.398,1.506)	1.416***	(1.400,1.420)	1.274***	(1.266,1.298)	1.117***	(1.100,1.207)	1.775***	(1.700,1.805)
Physiotherapy-cost	1.186***	(1.109,1.190)	1.065***	(1.054,1.070)	2.902***	(2.900,2.912)	2.889***	(2.880,2.909)	1.725***	(1.720,1.743)
Speech therapy-cost	1.026***	(0.978,1.342)	1.817***	(1.619,1.817)	1.967***	(1.890,2.070)	2.129***	(2.109,2.234)	1.725***	(1.712,1.730)

**Note:*τ=*quantile,*β***_***j=***_coefficient, 95%CI = 95% confidence interval, *p < 0.05, **p < 0.01, ***p **< 0.0001. All coefficients are per 5-year period**

Figure 1 above presents a concise visual summary of the estimated quantile regression results of the stroke direct costs predictors. Each plot illustrates the effect of one of the explanatory variables in the quantile regression model. The dash-dot-dot curve with filled dots represents the 19 point estimates of the coefficient for ’s ranging from 0.05 to 0.95. In this regard, *τ* is representing different quantiles. The shaded grey area shows a 95% pointwise confidence band. Superimposed on the plot is a solid red line representing the ordinary least squares of the mean, with two dashed red lines representing a 95% confidence interval for a particular coefficient [14]. Each plot is showing different quantiles on the *x-*axis and the covariate effect on the *y-*axis. Results illustrated in each plot are similar to those shown in Table 4. The results have shown that QR models provided much more information about the underlying associations better than the ordinary least regression, suggesting that the conditional distribution of stroke direct costs did not only differ by their means but also by their lower and upper tails. Thus, the ordinary least squares regression missed critical aspects of the associations that exist between the conditional distributions of the stroke direct costs and its predictors as shown in Fig. 1. For instance, the impact of age group 55–75 years on stroke direct costs were higher at the upper quantiles and lower, at the lower quantiles of the stroke direct costs. Further, the impact of female gender on stroke direct costs was higher at the lower and upper quantiles of the distribution of the costs. The magnitude of the association for physiotherapy cost increases from low to high quantiles. This result implies that the physiotherapy cost effect on stroke direct costs was much larger at the upper end (extremes) than the lower and central locations.

## Discussion

This study identified and quantified predictors of stroke direct costs in SA, using quantile regression to elucidate the differential effects of each putative predictor on stroke direct costs. The important drivers of stroke direct costs were the female gender, higher age groups, Indian/Asian, and black races, hypertension, cholesterol, heart problems, diabetes, medication, inpatient/hospitalisation, outpatient, physiotherapy, and speech therapy costs. The study results have shown that quantile regression models provided richer information about the underlying associations better than the ordinary least squares regression, signifying that conditional distributions of stroke direct costs did not only differ by their means but also by their lower, central location and upper tails as reported in previous studies. Diringer et al. [20] also found old age, heart problem, male sex, the severity of the stroke, and hospitalisation as predictors of ischemic stroke hospital costs. Similar findings were revealed by a study done in the UK, which found that the mean stroke costs were higher in females aged between 75 and 84 years [21]. A study conducted in the Netherlands established that hospitalisation costs contributed a larger proportion to total stroke costs and age was significantly associated with stroke costs [22]. A Korean study concluded that elderly men, inpatient and outpatient cost contributed the highest proportion to direct costs of stroke [23]. Stroke costs were found to be higher in men than those in women ([23, 24]. The study broadly supports earlier studies that investigated risk factors of stroke costs in developing countries. Elderly people, females, blacks and factors such as hypertension, cholesterol, heart problems, and diabetes increased the risk of stroke. Further, black South Africans had a higher impact on stroke direct costs than whites. Reducing the burden of stroke in the SA population requires identification of non-modifiable risk factors of stroke and demonstration of the efficacy of the risk reduction effort and impact on costs. Since the risky gender group has been identified in SA, this study recommends campaign services on raising awareness of the dangers of stroke risk, targeting the female gender and riskier groups.

Apart from non-modifiable factors being significantly associated with stroke direct costs, modifiable predictors such as hypertension, cholesterol, heart problems, and diabetes were also found to be significantly associated with stroke direct costs. The mean stroke medical costs were found to be higher in men aged ±68.8 years with hypertension and cholesterol [25]. Wang et al. [24] found that hypertension and diabetes were associated with higher costs. A Lebanese study identified the cost drivers for stroke direct medical cost as the severity of the stroke, length of stay in hospital (LOS), male sex, hypertension, cholesterol, diabetes, Atrial fibrillation, and smoking [25].

A study conducted in Finland also found that elderly females with hypertension, heart disease and diabetes were more expensive than men [26]. Largely comorbidities such as hypertension, cholesterol, heart problems, smoking, and diabetes are the major drivers for stroke direct costs. The study recommends regular screening and testing and treatment of hypertension, cholesterol, heart problems and diabetes in SA to detect the risk of stroke early enough.

The study has several important findings, hospitalisation, medication, and physiotherapy costs contributed a larger proportion to total stroke direct costs in SA. A Danish study also found the attributable costs of direct net health care services after stroke as age, gender, general practitioner services, hospital services, and medication [27]. Saka et al. [28] found that larger proportions for stroke direct costs in the UK were from diagnosis costs, inpatient care costs, outpatient care costs, and drug costs. Inpatient treatment and medication costs significantly contributed to the health care costs for stroke patients [27]. Numerous studies found, LOS, intensive care unit, stroke severity, old age, male gender and comorbidities such as hypertension, cholesterol, diabetes and heart problems as the major drivers of the direct medical cost of hospitalisation for stroke [24, 25, 29]. Overall, inpatient costs, outpatient costs and medication costs were identified as the major contributors to stroke direct medical costs. The finding that the proportion of hospitalisation cost was higher than other proportions of stroke cost items is consistent with previous studies [12, 25].

There were some limitations to this study. Risk factors such as stress, HIV/AIDS, smoking and alcohol consumption were not captured in the patient’s records. Clinical severity factors such as neurologic severity were not available in the data, yet it is an important predictor of costs. The indirect costs due to loss of productivity and family members’ informal care were not examined since the data set did not include this information. Further studies could consider including all risk factors of stroke and examining the indirect costs for a more comprehensive evaluation of stroke costs. Nevertheless, the strengths of this prevalence-based cost of illness study lie in that all cases of stroke are included for the specific period 2014 to 2018. Also, recent data set has been used without missing information. To date, this is the only comprehensive QR study on modelling the direct costs of stroke in SA.

## Conclusion

This study provides useful insights for healthcare programs and policies on the risk of hypertension, cholesterol, heart problems and diabetes in SA. The significance of comorbidities of stroke should be examined further to identify and treat groups at high risk of developing stroke so that its costs and consequences for patients and families can be reduced in SA. It further highlights the need for more improved health awareness efforts to increase knowledge of modifiable stroke predictors in the South African population, and to support healthy life-style choices among South Africans. Thus, reporting the average costs of stroke without an account of these factors may be pointless when assessing the cost-effectiveness of stroke interventions. More broadly, we hope that our detailed cost information on stroke patients could be used to develop cost-effective programs for preventing this epidemic while simultaneously reducing the stroke economic burden.

The estimated cost is expected to provide an evidential source for use in determining cost-effective policies on management stroke and making plans for arranging medical sources as well as to determine priorities for the effective prevention of stroke and national healthcare policies. Although stroke is a disease of ageing, we found a high prevalence of stroke in relatively young people. This is worrying because a large proportion of productive working age group is affected in SA, we recommend further studies to understand the reasons for the new trend of relatively young strokes. The identified predictors can aid the formulation of healthcare policies and early interventions that will improve stroke outcomes and reduce costs. Thus, this study calls for early interventions and treatment of hypertension, cholesterol and diabetes in SA. The identification of more vulnerable groups can be used to formulate more effective interventions for these groups. Finally, the use of quantile regression modelling technique is recommended for identifying and quantifying stroke direct costs predictors because quantile regression leads to more comprehensive results because of its ability to assess the effect of each predictor at any part of the outcome distribution.

## Abbreviations

YLL: Years productivity and years life lost
NCD: Non-communicable diseases
LMIC: Low and middle-income country
YLD: Years lived with a disability
UK: United Kingdom
LOS: Length of stay in hospital
CT: Computed tomography
MRI: Magnetic resonance image
HDL: High-density lipoproteins
LDL: Low-density lipoproteins
OLS: Ordinary least squares
QR: Quantile regression
CI: Confidence interval
MCMC: Markov Chain Monte Carlo
SSA: Sub-Saharan Africa
SA: South Africa
